# Medical students in their first consultation: A comparison between a simulated face-to-face and telehealth consultation to train medical consultation skills

**DOI:** 10.3205/zma001645

**Published:** 2023-09-15

**Authors:** Lena Dahmen, Maike Linke, Achim Schneider, Susanne J. Kühl

**Affiliations:** 1 University of Ulm, Faculty of Medicine, Institute of Biochemistry and Molecular Biology, Ulm, Germany; 2 Dresden University of Technology, Carl Gustav Carus Faculty of Medicine, Psychosocial Medicine and Developmental Neurosciences, Dresden, Germany; 3 University of Ulm, Faculty of Medicine, Dean of Studies Office, Ulm, Germany

**Keywords:** communication between the physician and a patient’s family members, simulated conversation, actors as patients, biochemistry, e-learning

## Abstract

**Objective:**

A simulated conversation between a physician and a family member, i.e., a medical conversation, was changed from a conventional face-to-face conversation (SS 2019) to a telehealth conversation (SS 2020) due to the COVID-19 pandemic. The medical education conversation is part of the biochemistry seminar “From Genes to Proteins” which second semester human medicine students take. The objective of this study was to analyze to what extent the switch from face-to-face to telehealth conversations affected student satisfaction and motivation.

**Methodology:**

In the seminar, students study biochemical as well as competency-oriented content, such as how to talk to family members. In the summer semester of 2019, students were trained how to talk to their patients’ family members in a traditional conversation setting with the help of lay actors in a classroom format. In the summer semester of 2020, this conversation took place under comparable conditions, but in the form of an online telehealth conversation instead. Student satisfaction and motivation were surveyed by means of an evaluation questionnaire following the seminar in both semesters.

**Results:**

Both conversation formats achieved a high level of satisfaction from students (school grade A-B). For some evaluation items, such as “realistic conversation simulation”, the face-to-face conversation was perceived as more satisfying (*Md=5.0, IQR=1.0*) than the telehealth conversation (*Md=5.0, IQR=2.0*). In addition, the face-to-face conversation resulted in higher subjective motivation from students (*Md=5.0, IQR=1.0*) than that of the telehealth conversation (*Md=4.0, IQR=2.0*).

**Conclusion:**

The high student satisfaction and acceptance of both didactic concepts leads to the conclusion that the simulated telehealth conversation is an adequate substitute for the simulation of a traditional face-to-face conversation with regard to the parameters that were studied.

## Zusammenfassung

**Zielsetzung:**

Das simulierte Arzt-Angehörigen-Gespräch im Sinne einer ärztlichen Sprechstunde wurde aufgrund der COVID-19-Pandemie von einer konventionellen Präsenz-Sprechstunde (SS 2019) zu einer Tele-Sprechstunde (SS 2020) umgestellt. Das ärztliche Aufklärungsgespräch ist Teil des Biochemieseminars „Vom Gen zum Protein“, an welchem Studierende der Humanmedizin im 2. Semester teilnehmen. Ziel dieser Studie war zu analysieren, inwiefern sich die Umstellung auf die Zufriedenheit und Motivation der Studierenden auswirkte.

**Methodik:**

Im Seminar erlernen die Studierenden neben biochemischen auch kompetenzorientierte Lerninhalte, wie die ärztliche Gesprächsführung im simulierten Arzt-Angehörigen-Gespräch. Im SS 2019 fand das Training der ärztlichen Gesprächsführung mit Laienschauspieler*innen als traditionelle Sprechstunde im Präsenzformat statt. Im SS 2020 wurde das Arzt-Angehörigen-Gespräch unter vergleichbaren Bedingungen, jedoch als Tele-Sprechstunde, durchgeführt. Die Zufriedenheit und Motivation der Studierenden wurde durch einen Evaluationsbogen im Anschluss an das Seminar erfragt.

**Ergebnisse:**

Beide Sprechstundenformate erzielten eine hohe Zufriedenheit seitens der Studierenden (Schulnote 1-2). Bei einigen Evaluationsitems, wie beispielsweise der „realitätsnahen Sprechstundensimulation“ erzielte die Präsenz-Sprechstunde eine höhere Zufriedenheit (*Md=5,0, IQR=1,0*) im Vergleich zur Tele-Sprechstunde (*Md=5,0, IQR=2,0*). Zudem konnte die subjektive Motivation der Studierenden im Medizinstudium durch die Präsenz-Sprechstunde (*Md=5,0, IQR=1,0*) stärker gesteigert werden als durch die Tele-Sprechstunde (Md*=4,0, IQR=2,0*).

**Schlussfolgerung:**

Die hohe Zufriedenheit und Akzeptanz der Studierenden für beide didaktische Konzepte lässt schlussfolgern, dass die simulierte Tele-Sprechstunde bezüglich der untersuchten Parameter einen adäquaten Ersatz für die Simulation einer traditionellen Präsenz-Sprechstunde bietet.

## 1. Introduction

### 1.1. Background

Interpersonal communication is an important component of any relationship. Consequently, it is also the basis of a good relationship between physicians and their patients [7]. Both verbal and nonverbal communication has a significant impact on the success of any treatment. The ability of a physician to show empathy when talking to patients or family members, for example, as well as open body language, e.g., uncrossed legs and arms, are associated with a positive treatment outcome [1].

The interaction between a physician and a patient not only influences the rapid and correct diagnosis and the course of treatment, but also the patient’s compliance (willingness to cooperate), satisfaction and quality of life [1], [2], [5], [18], [28]. Conversely, some types of behaviors, such as an inappropriately formal or dominant behavior by a physician, can also lead to negative therapeutic effects [1]. Any medical conversation with the family members of a patient is also of particular importance, because family members are considered an important support system for patients and also influence the progression of the illness, compliance, and thus the course of treatment [31].

These positive and negative effects show the relevance of a good relationship between the physician and the patient and their family members, since they are closely associated with good patient care. Therefore, it is very important that students in human medicine (and thus, future physicians) are taught good conversational skills. These skills can help communicate complex issues to people who are not specialists in the field in a compassionate and comprehensible manner. Communication skills should therefore be taught and trained as part of their studies. Some universities teach medical communication as a longitudinal curriculum, which is often a required class [8], [10], [12], [13], [21], [32]. In German-speaking countries, (lay) actors are increasingly used in role plays designed for this purpose [10], [25].

### 1.2. The initial situation in the biochemical seminar at the University of Ulm and the objective of the study

The integrated biochemical seminar “From Genes to Proteins” is offered during the preclinical phase of human medicine studies at the University of Ulm and pursues biochemical and competency-oriented learning objectives [4], [16], [23].

A previous study had shown that the conversion of the biochemistry seminar from an inverted classroom (IC) concept to an online-only seminar was very successful in terms of student satisfaction and acceptance, as well as exam results, demonstrating the online format as an adequate substitution to in-person learning [4]. One part of the seminar relates to competency-based learning objectives, during which students also learn how to have a conversation with family members by using simulated scenarios [4], [16], [23].

In 2019, the simulated conversation with family members was conducted as a traditional face-to-face conversation, while in 2020, in the wake of the COVID-19 pandemic and associated contact restrictions, it was conducted as a telehealth conversation.

The switch from face-to-face to telehealth conversation provided the opportunity for a direct comparison of the two formats in the same seminar under comparable conditions. In order to determine whether and to what extent the switch influences the satisfaction, interest and motivation of students, the conversations were analyzed and compared.

The objective of this study was to compare student satisfaction, interest, and motivation as a result of a simulated conversation between a physician and family members using a traditional face-to-face format and a telehealth format. 

For this purpose, the following specific questions were investigated:

1. Is student satisfaction regarding the preparation, implementation, and performance of a simulated medical education conversation in the face-to-face format comparable to that in a telehealth format?
2. Does the format, i.e., either face-to-face or telehealth, affect the students’ subjective motivation in medical school and interest in biochemistry?
3. For which aspects of the simulated conversation do students express praise, criticism or a need for improvement?

## 2. Materials and methods

### 2.1. Course description

The study was conducted as part of the integrated biochemistry seminar “From Genes to Proteins” at the Medical Faculty of Ulm in the preclinical study phase. In the seminar, second semester students of human medicine study biochemical and competency-oriented learning objectives in 16 seminar groups, with a group size of about 20 students each. The same two lecturers led the seminar both times [4], [16], [23]. The seminar was offered during the summer semester (SS) 2019 and used the IC concept (alternation between asynchronous online-based self-learning phases and synchronous physical presence phases at the university), while in SS 2020 it was taught using an online-only teaching concept (alternation between asynchronous online-based self-learning phases with synchronous online presence phases). Both seminars were identical in terms of content; the only differences were the didactic structure and implementation. A detailed schedule of the seminar as well as further information on the implementation of the seminar in 2019 and 2020 can be found in our previous study [4].

### 2.2. Training of medical conversation skills

To practice medical conversation skills, students practiced a simulated conversation with family members during the respective (online) presence phase in 16 seminar groups. They were provided with a worksheet outlining basic medical conversation skills as well as detailed scenarios for the role of the physician and the role of the observers in advance (in the classroom or via the Moodle learning platform). Furthermore, they received information about a clinical case of a patient with *osteogenesis imperfecta* as well as an X-ray and information about a family member of the patient [4]. Prior to the simulation exercise, the students in a given seminar group were randomly divided into two teams, an “observer team” and a “physician team”. The “observer team” was further divided into students providing feedback and observers. One student from the physician team took on the role of the physician and, together with the rest of the team, used the scenario description to prepare for the conversation. The observer team prepared feedback in a self-organized manner. Also present were a lay actor in the role of the patient’s family member, and the lecturer. The conversation between the physician and the family member was simulated one time in each seminar group as part of an (online) class. After the simulated conversation had taken place, the physician group was provided with feedback and the conversation was discussed on the basis of an evaluation form. First, the student who had played the doctor was asked to talk about how they felt about the conversation. Then, the lay actor, the students providing feedback, the observers, as well as the lecturer gave their feedback [4].

#### 2.2.1. The lay actors in the role of family members

The actors who assisted with the simulated conversation during the summer semester 2019 were students enrolled in the Actor-Patient Program of the Medical Faculty Ulm. The curriculum includes general role plays, role-specific training and feedback workshops [25]. The objective of the program is to train lay actors in scientific and communicative aspects in order to be well prepared for the conversation between the physician and the family member and be able to reflect on the interview in a professional manner [25]. For the simulated conversation during the summer semester 2020, two female staff members of the Institute of Biochemistry and Molecular Biology were recruited and trained, because the online semester had to be organized very quickly and in a short period of time due to the COVID-19 pandemic. Due to this time limitation and contact restrictions, this was the only possible way to conduct the conversation in a telehealth setting. The two employees were introduced to the role of family member in a manner similar to that of the lay actors during the summer semester 2019. The scenario used during the summer semester 2020 was only adapted to the online situation; in all other aspects, it was identical to that used during the summer semester 2019.

Both times, actors assumed the role of a concerned family member during a conversation with the physician in order to ask the students in the physician role about the patient's illness. For more detailed information, please refer to our previous study [4].

#### 2.2.2. The face-to-face conversation during the summer semester 2019

During an initial in-person phase, the students were provided with a worksheet outlining basic medical conversation skills, as well as some information about their particular case. During the subsequent self-learning phase, the students were provided with the scenario for the physician’s role as well as the role of students providing feedback and acting as observers. In preparation for the next in-person phase, the students were asked to read the scenario and consider how they would conduct a conversation with a family member of their own. For this purpose, the students were asked to interview their friends and family to find out what their ideas were with regard to that particular conversation and its content. The simulation of the approximately 10-minute conversation between the physician and family member took place during in-person classes at the university following a short preparation phase. During this preparation phase, the students had the opportunity to review their patient’s history and the corresponding X-ray, as well as to use the teaching materials relating to medical conversation skills. The completed medical conversation was followed by a feedback discussion.

#### 2.2.3. Implementation of the telehealth conversation during the summer semester 2020

To prepare for the telehealth conversation, the students first received a document outlining basic medical conversation skills as well as the scenarios for the physician role and the role of the feedback givers and observers via the Moodle learning platform during the preceding self-learning phase.

During the subsequent online phase, the conversation between the physician and a family member was conducted in 16 seminar groups of approximately 20 participants each using the Big Blue Button video conferencing system. The students were informed in advance via Moodle about how the simulation and following discussion would be conducted. After entering the video seminar and activating the microphone and camera, the lecturer welcomed the students and instructed them how to use the technology. All students were instructed to pin only the two people in the simulation, the student in the physician’s role and the lay actor, for the subsequent simulation, so that only those two could be seen and heard. During the approximately 10-minute simulated conversation, the student in the role of the physician explained to the lay actor in the role of the family member what osteogenesis imperfecta is and what the treatment options are. The conversation was followed by a feedback discussion comparable to the face-to-face conversation during the summer semester 2019, during which the entire seminar group was able to actively participate via cameras and microphones.

### 2.3. Study design

**Figure 1:**
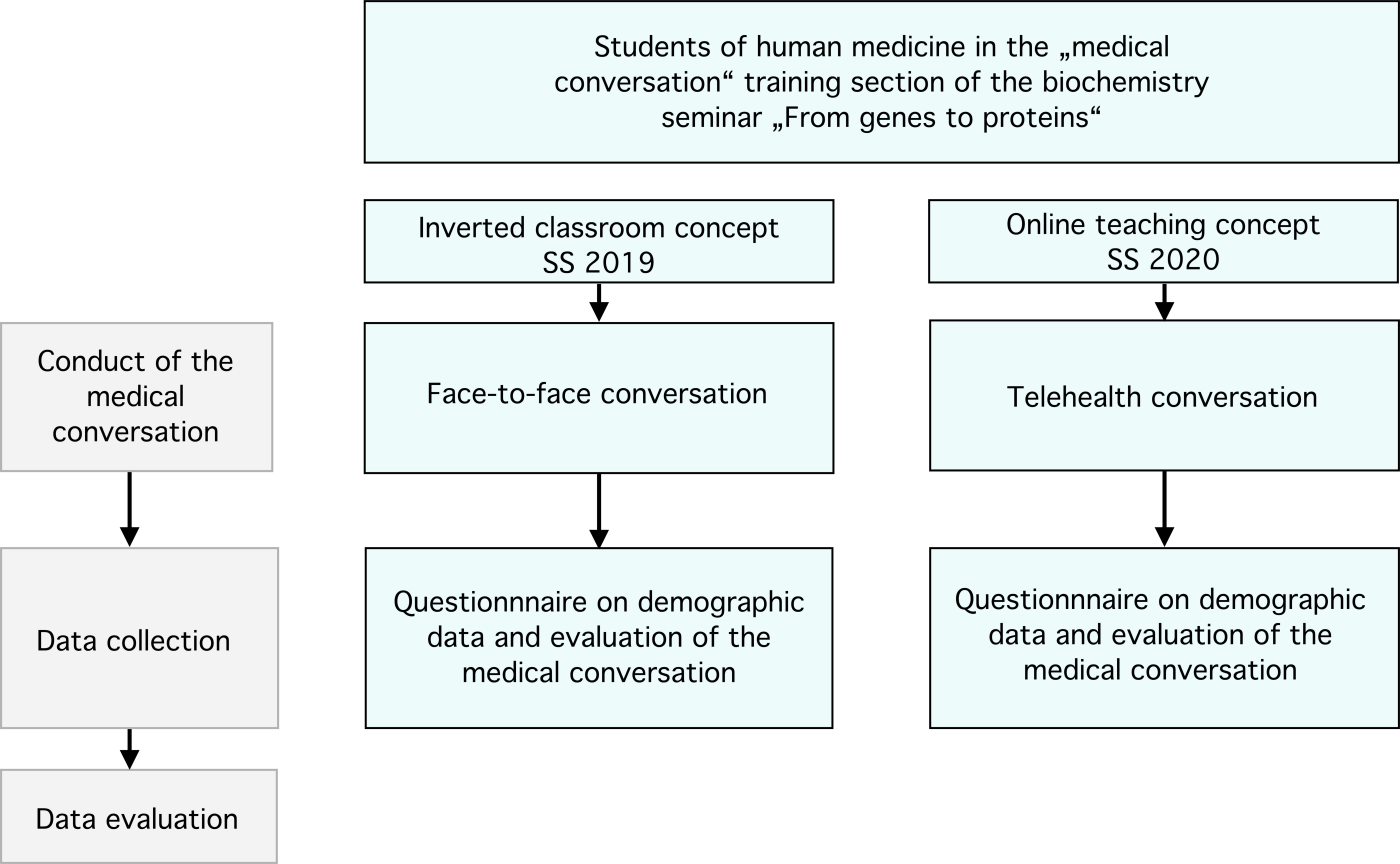
Study design The study design compares the two conversation formats. In the inverted classroom concept, which was used for the summer semester (SS) 2019, the conversation between the physician and a family member was carried out in a face-to-face format and as a telehealth conversation in the online learning concept used for the summer semester 2020. In 2019, 335 students participated in the study, followed by 322 in 2020. 164 evaluations were received in SS 2019 and 100 were received in SS 2020.

The purpose of this study was to compare the face-to-face conversation using the IC concept with the telehealth conversation using the online-only teaching concept (see figure 1 (Fig. 1)). The conversation between the physician and a family member was evaluated both times using a comparable evaluation form from the Institute of Biochemistry and Molecular Biology (see attachment 1 ). 335 students attended the seminar in the summer semester 2019, followed by 322 students in the summer semester 2020. 164 students (49% of the seminar participants) completed the simulation evaluation form in 2019 and 100 students (31.1% of the seminar participants) completed the form in 2020.

### 2.4. Data acquisition

#### 2.4.1. Quantitative and qualitative data collection

Both in 2019 and 2020, an anonymous and voluntary evaluation (see attachment 1 ) was conducted immediately after the seminar. It collected the following information:

##### Demographics

To compare the two study groups, students were first asked questions about themselves. The students provided their age and gender. Furthermore, they were asked about their previous training in the medical field (total duration>1 year) and university studies relating to the medical field (total duration>1 year).

##### Evaluation of the conversation between the physician and a family member

The questionnaire used to simulate the conversation between the physician and a family member consisted of 12 questions. Questions 1-4 pertained to an interest in biochemistry and motivation in medical school before and after the simulated conversation. Questions 5-11 covered the preparation, implementation, and performance of the conversation between the physician and a family member. Questions 1-11 used a Likert-type response scale ranging from 1 (strongly disagree) to 6 (strongly agree). Students were also given the option to select the answer “N/A” for any of the questions. Question 12 asked students to grade the simulated conversation, ranging from A (very good) to F (fail). Most of the questions evaluating the face-to-face conversation had been successfully used in one of our previous studies and published [25]. The questions about the telehealth conversation were newly created in the context of the present study, but only the wording of the questions was adapted to the online conversation format, but nothing was changed in terms of content. Thus, a 1:1 comparison of the two didactic concepts was possible.

##### Free text fields

In an additional field, students had the opportunity to provide free-text comments under the two headings of praise and criticism or to make specific suggestions for improving the simulated conversation and its preparation. 

#### 2.4.2. Statistical analysis

Since the Kolmogorov-Smirnov test showed that the data was not normally distributed (*p*<0.05), the Wilcoxon-Mann-Whitney U test was used to compare the results of the evaluation. A *p value* of *p*<0.05 was considered significant. The effect sizes were calculated using* r* (r=z/square *root n*). The IBM SPSS Statistics program, version 26 for Mac OS was used for the data analysis [4].

The chi-square test was used to analyze the demographic data and the quantified free text comments. The free text comments were quantified by counting positive and negative comments or comments with suggestions for improvement. Comments that contained both positive and negative comments or comments with suggestions for improvement were not counted because the identification of clearly positive or clearly negative comment content was difficult to assess and the direct comparison of praise and criticism could be presented more clearly without them. This procedure had already been successfully used for our previous study [23]. Comments that did not refer to the performance of the conversation were not included either. An excerpt of comments with frequently mentioned praise or improvement suggestions is provided in the results chapter 3.4.

### 2.5. Ethics

The ethics committee of the University of Ulm confirmed in writing that an ethics application was not necessary for this study. The data was provided voluntarily and anonymously, and the students were not compensated for their participation. Furthermore, the students were informed that by submitting the evaluation form, they agreed to data processing.

## 3. Results

### 3.1. Comparison of student demographics in SS 2019 and SS 2020

The demographic data of the participants were examined to compare the two study groups. In addition to their gender and age, the students also listed their previous education in terms of having already completed their training or studies. The data show that there was no significant difference between the two study groups in terms of demographic data (see table 1 (Tab. 1)).

### 3.2. Analysis of student satisfaction due to the different simulation formats

Students rated both the face-to-face and the telehealth conversation with a comparable overall grade of A-B (“I give the following grade to the simulated conversation including preparation”, ***SS 2019: ****median (Md)=5.0 (corresponds to grade B), interquartile range (IQR)=1.0, n=164; ****SS 2020:**** Md=6.0 (corresponds to grade A), IQR=1.0, n=98). *

**Figure 2:**
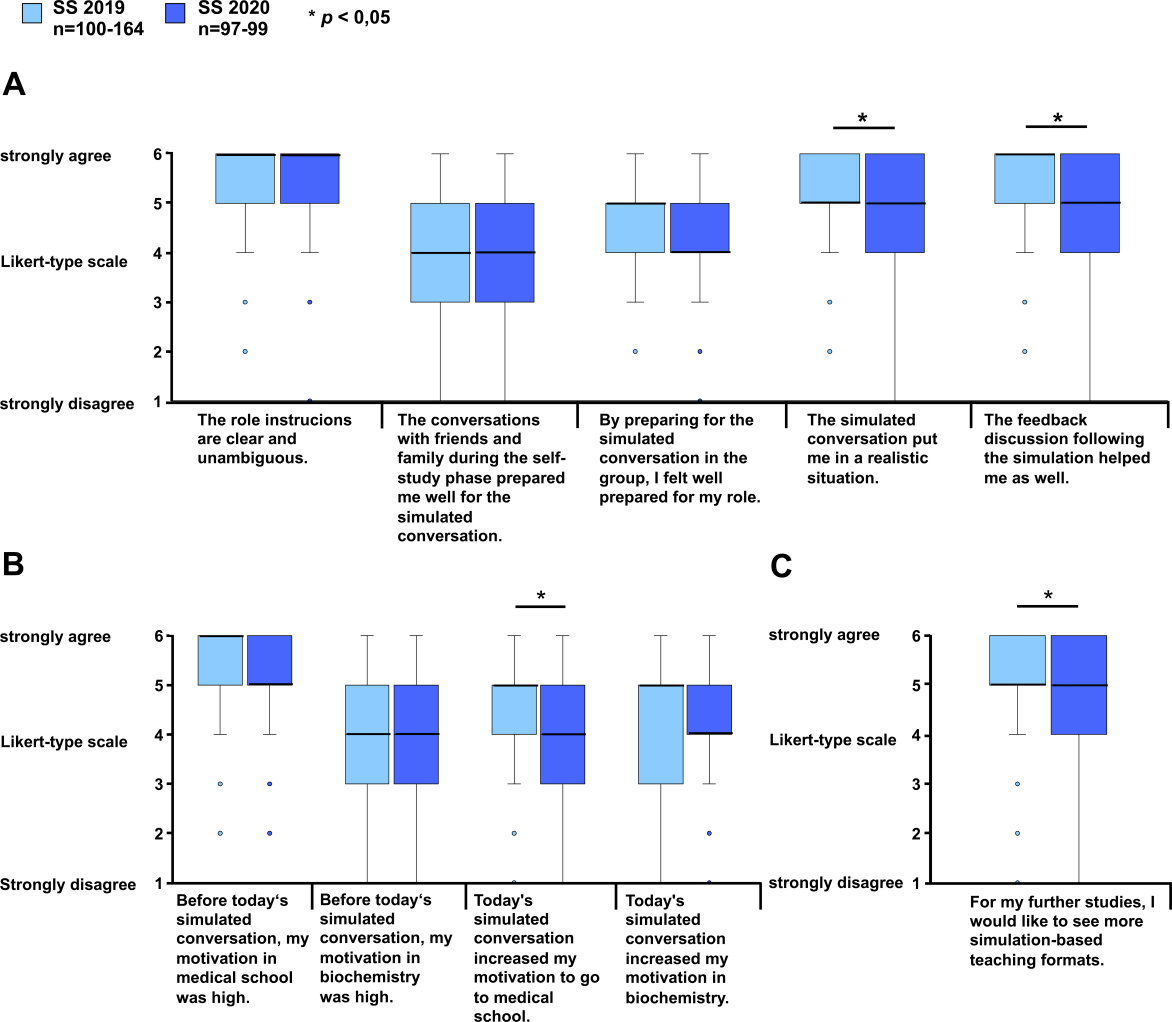
Evaluation results Comparison of the evaluation results for the face-to-face and the telehealth conversation. The students evaluated the two conversation formats by using a Likert-type response scale ranging from 1 (“strongly disagree”) to 6 (“strongly agree”). A. Evaluation of the conversation. B. Motivation to go to medical school and interest in biochemistry. C. Student desire for simulation-based formats throughout their studies. n=number of participants

The traditional face-to-face conversation scored significantly higher in terms of the simulation. Both the realistic simulation of the conversation between the physician and a family member (“The simulated conversation put me in a realistic situation”, ***SS 2019:**** Md=5.0, IQR=1.0, n=151; ****SS 2020:**** Md=5.0, IQR=2.0, n=97; p=0.0003, r=0.2)*, as well as the feedback discussion (“The feedback discussion following the simulation helped me as well” ***SS 2019:**** Md=6.0, IQR=1.0, n=156; ****SS 2020:**** Md=5.0, IQR=2.0, n=98; p=0.0002, r=0.2*) were rated significantly better by students who used the face-to-face format than those who used the telehealth format. Additionally, there was a significant difference regarding the desire for an increased implementation of simulation-based teaching formats in further studies (“For my further studies, I would like to see more simulation-based teaching formats” (***SS 2019:**** Md=5.0, IQR=1.0, n=162; ****SS 2020:**** Md=5.0, IQR=2.0, n=99; p=0.000004, r=0.02*). The effect size r of significant differences was small. Both formats achieved comparable results in all other areas of the simulated conversation (see figure 2 (Fig. 2)).

### 3.3. Subjectively perceived student motivation and interest due to the different simulation formats

Furthermore, students were asked about their motivation to go to medical school and their interest in biochemistry. With regard to their motivation to go to medical school prior to the respective simulated conversation, both formats achieved comparable results. For the subjectively perceived motivation to go to medical school after the simulation, students showed a significant increase due to the face-to-face conversation compared to the telehealth conversation (“Today’s simulated conversation increased my motivation to go to medical school”, ***SS 2019:**** Md=5.0, IQR=1.0, n=159; ****SS 2020:**** Md=4.0, IQR=2.0, n=99; p=0.00008, r=0.2*). The effect size r can be described as small. Student interest in biochemistry prior to and after the simulated conversation, was evaluated in terms of the different formats as well (see figure 2 (Fig. 2)).

### 3.4. Analysis of the free text comments regarding the different conversation formats

An analysis of the free-text comments revealed that the face-to-face conversation in SS 2019 provided students with a realistic and motivating glimpse into the future. Students noted that they would like to learn more about how to conduct such conversations.

In the comments about the online seminar in SS 2020, the students praised the commitment of the lecturers and the exercise that taught them how to describe complex issues in simple terms. One student suggestion was to conduct several short simulations with fewer students. Students also addressed some technical problems with the implementation of the telehealth conversation.

A quantitative analysis of the comments showed a small decrease in positive comments (praise) from 85% in SS 2019 to 80% in SS 2020. The comments with suggestions for improvement increased from 15% in SS 2019 to 20% in SS 2020. The chi-square test showed that there was no statistically significant difference between the teaching methods in terms of the number of positive comments (praise) or negative comments (criticism/suggestions for improvement) (*p*=0.35). In SS 2019, 14% of all the comments submitted contained both praise and criticism/improvement suggestions, while in SS 2020, 27.6% contained both. Comments that did not pertain to the simulated conversation and were therefore not included in this study accounted for 9.9% of the total comments submitted in SS 2019 and 3.4% in SS 2020 (see table 2 (Tab. 2)).

## 4. Discussion

This study shows that conversation between a physician and a patient’s family member conducted in a telehealth format is, for the most part, a good substitute for face-to-face conversation. Student satisfaction and motivation were taken into account.

### 4.1. Student satisfaction was comparable in both formats

The results of our evaluation show that both face-to-face and telehealth conversation achieved high levels of satisfaction from students. The three significant results in the area of the simulated conversation have a small effect size and thus represent small deviations in student satisfaction. Other studies also show that training for a conversation between a physician and a family member in the form of a simulated conversation has already been implemented with great student approval [10], [32]. An online class to provide communication training has already been successfully implemented at some other medical schools with mostly positive feedback from the students [9], [11], [14], [17], [20], [29]. Furthermore, the switch of a communication course from an in-person to an online format was studied at the University of Frankfurt, Germany. The course covered topics such as communication theories and questioning techniques as well as a simulated medical history interview [26]. The results of that study show a high level of participant satisfaction or agreement in terms of the subjectively perceived growth in learning, the relevance of communication and conversational skills as well as the atmosphere in the course. Most of the results from that study were similar to the results of in-person courses conducted previously. Significant differences were found with regard to the course structure. Students did not feel that the online format is suitable for teaching how to take a patient’s medical history [26]. Especially the positive evaluation of the atmosphere during the course and some results of the in-person and online formats e.g. “preparation for a medical conversation” reflect the tendency of our data. 

### 4.2. Students feel more motivated and interested as a result of the simulated conversation 

The students who participated in our study indicated that their motivation to go to medical school and their interest in biochemistry was subjectively increased by the simulated conversation in both the face-to-face and telehealth format. Contact with patients during the preclinical phase also significantly increased student motivation in basic subjects at the University of Essen [22]. By having contact with family members during our seminar, students get a first glimpse into the daily life of a physician very early in their course of studies and at the same time gain some experience about how to communicate with patients or their family members. Thus, it is understandable that the glimpse into the daily life of a physician has a subjectively motivating effect on the students and, accordingly, increases their interest in biochemistry at the same time. By directly using their knowledge in a conversation with laypersons, students learn how relevant and important the basic subjects are. They also learn how to explain complex issues to laypersons in a simple and understandable manner. This helps them not only recognize the importance of not only memorizing the lecture material as factual knowledge, but also to understand what they have learned so that they can subsequently “translate” it into easy-to-understand language. Another study showed that the students’ confidence in talking to patients was significantly increased by conducting a simulated doctor/patient conversation [30]. In future studies, it would be interesting to find out whether and to what extent their communication competence is measurably improved.

### 4.3. Predominantly positive comments about both formats

The option to add free text comments gave students the opportunity to provide feedback about the seminar independently of the evaluation questions asked. In another study, participants in a simulated physician/patient conversation also had the opportunity to provide written feedback [19]. Student comments in this study were consistently positive. Participants praised the realistic design of the physician conversation and the useful feedback, which supports our findings. Participants in our study stated, for example, that the simulated conversation provided good insight into clinical practice, making them feel better prepared. This experience is also shared by students from the UK, who felt better prepared for everyday clinical practice as a result of a simulated telehealth conversation [3]. 

In our study, technical issues were cited as a problem in implementing the telehealth conversation, particularly with regard to the microphones and sound. Scottish hospital personnel shared the same experience. In a study, they conducted online conversations with patients and found similar technical problems, such as the camera “freezing” or poor audio quality during the conversation [6]. It would therefore be interesting to analyze in further studies to what extent such problems can be eliminated through further technical improvements.

### 4.4. Strengths and weaknesses of the study

A strength of the study is the fact that the same two lecturers conducted the courses both times, regardless of the teaching method. In addition, both lecturers have comparable levels of teaching experience and motivation. Thus, it stands to reason that the teaching in both years and in all seminar groups differed primarily in terms of methodology, but not significantly in the didactic quality of the lecturers. Any differences in the evaluations from SS 2019 and SS 2020 thus essentially address the changed teaching method. 

A weakness of the study is the difference between the lay actors in SS 2019 and SS 2020. The lay actors in SS 2019 were part of the acting program of the Medical Faculty of Ulm, while the two female lay actors in SS 2020 were employees of the Institute of Biochemistry and Molecular Biology. Both times, however, the lay actors played the roles of a parent and a grandparent. Furthermore, care was taken to train the lay actors in a comparable manner both times by conducting a preliminary conversation, with the lecturer having the main responsibility (S.J.K).

Another weakness of this study is the different participation ratios in the two summer semesters 2019 and 2020. In SS 2019, 164 students participated in the evaluation of the face-to-face conversation (equivalent to 49% of the seminar participants). However, in SS 2020, 100 students participated in the evaluation of the telehealth conversation (equivalent to 31.1% of the seminar participants). 

One reason for the higher number of participants in SS 2019 could be that the students were provided with a printed evaluation form at the end of the seminar during the in-person phase at the university and were personally asked to complete it on-site. The SS 2020 students, on the other hand, evaluated the telehealth session online during an online learning phase, in which they were sent the evaluation form questionnaire via email. Similarly, a previous study that compared the IC and online teaching concepts in the same “From genes to proteins” teaching concepts also showed a lower participation rate in SS 2020 [4]. In that study, however, the participating students completed both the SS 2019 and SS 2020 evaluations online. Therefore, the lower participation rate in SS 2020 is more likely due to the study group and, as a result, presumably due to the cohort effect.

Furthermore, only the evaluation results of students were considered in this study. This study did not examine the extent to which the change in the teaching method had an effect on the acquisition of or increase in medical communication competency. The establishment of an objective examination format to test communication competency would be useful for this purpose.

## 5. Conclusion and outlook

In summary, student satisfaction is high and largely comparable for both face-to-face and telehealth conversation simulations. The simulation of a telehealth conversation is thus a good substitute for the traditional face-to-face conversation. Both formats also achieve comparable evaluation results in the areas of student interest and motivation, but the subjectively perceived increase in motivation to go to medical school is in favor of the face-to-face format. Going forward, an analysis of the acquisition of competencies relating to medical communication skills in both concepts would be useful. Furthermore, it would be interesting to see whether and to what extent the results of this study or the comparison of the inverted classroom concept with the online teaching concept can also be reproduced after the COVID-19 pandemic under standard conditions.

## Acknowledgement

We would like to thank the students for participating in the surveys.

## Competing interests

The authors declare that they have no competing interests. 

## Supplementary Material

Questionnaire

## Figures and Tables

**Table 1:**
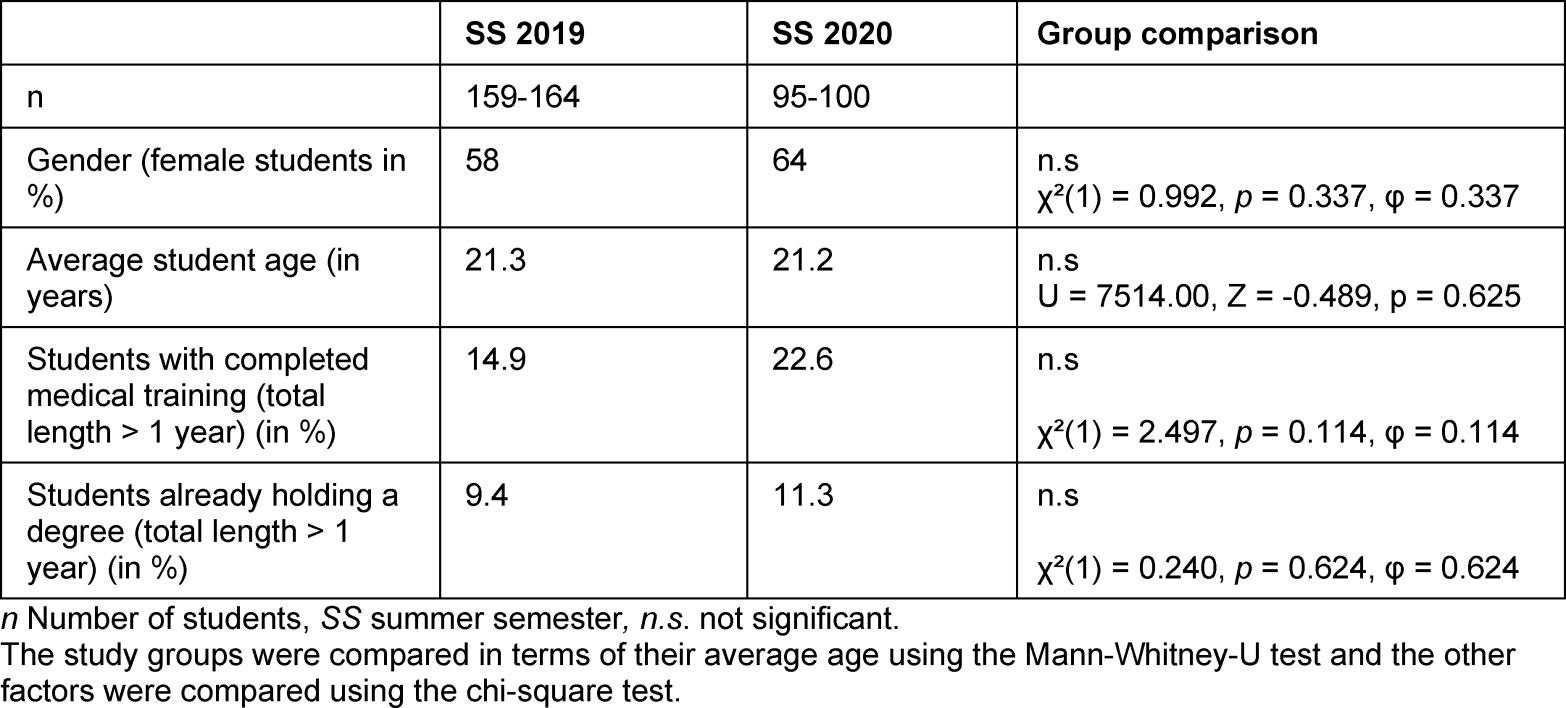
Study group comparison

**Table 2:**
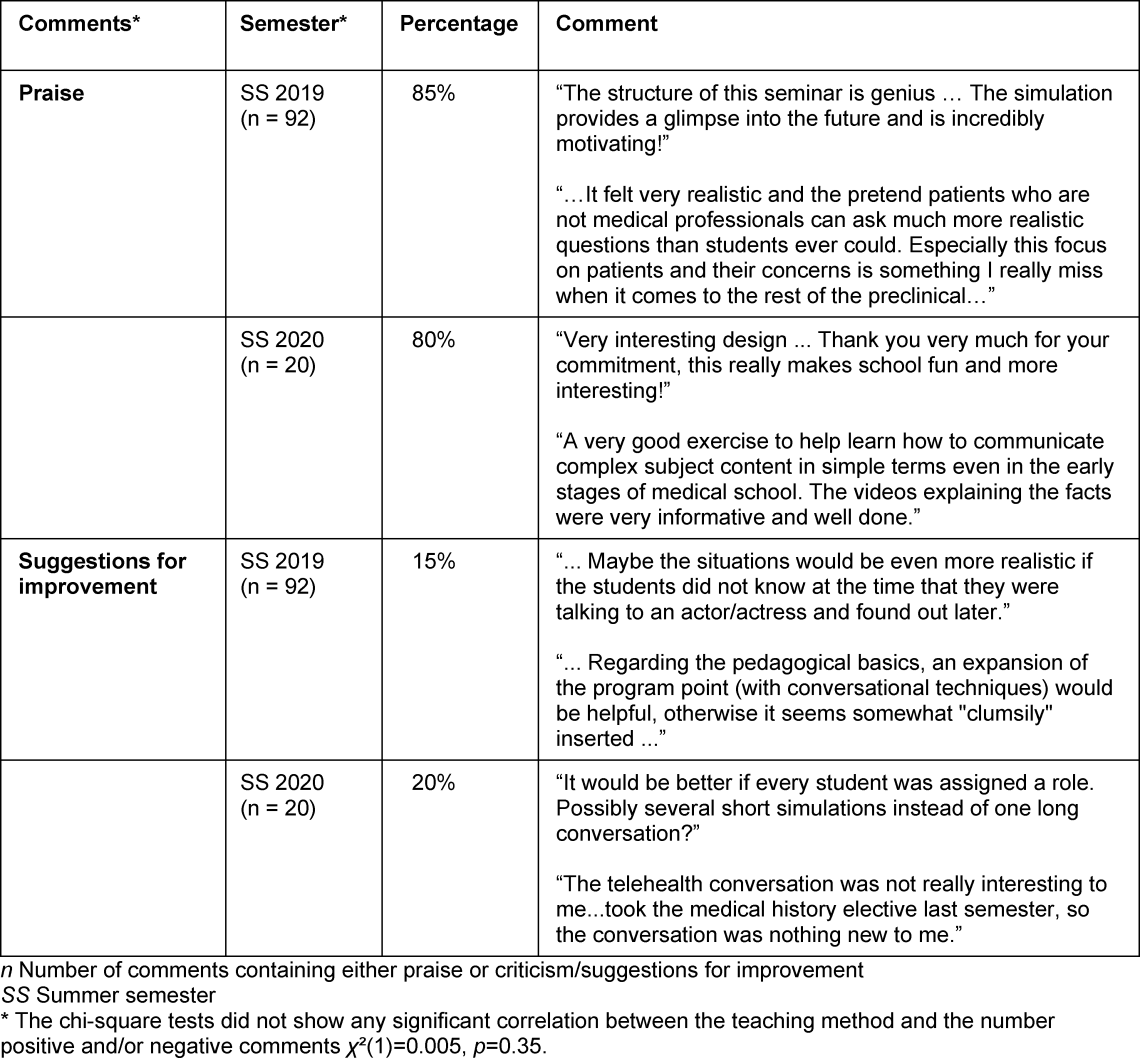
Comments by the participants
