# Supplementary material for: Medical students in their first consultation: A comparison between a simulated face-to-face and telehealth consultation to train medical consultation skills
Source: GMS J Med Educ. 2023 Sep 15;40(5):Doc63. doi: 10.3205/zma001645 (PMC10594035; doi:10.3205/zma001645)
Supplement: Questionnaire [file JME-40-63-s-001.pdf]

Attachment 1: Questionnaire

The differences between the questionnaires are highlighted for SS 2019 (IC) and SS 2020 (online teaching concept) in accordance with the different colors. Questions that are not marked were identical in both years.
Simulation evaluation questions in *italics* and bold were used in our previous study and published [25].

Data processing consent and general information

- **yes/no**

General information

- Age (free text)
- Gender
  Female/male
- I have already completed training in a related medical field (with/without working in the profession) (total duration >1)
  Yes/no
- I already have a degree from a university (total duration >1)
  Yes/no

Simulation

The evaluation is based on a Likert-type response scale from 1 (strongly disagree) to 6 (strongly agree).

- Prior to today‘s simulated conversation/the telehealth conversation, I was highly motivated to go to medical school.
- Prior to today‘s simulated conversation/the telehealth conversation, I was very interested in biochemistry.
- ***Today‘s simulated conversation/***the telehealth conversation ***increased my motivation to go to medical school.***
- ***Today‘s simulated conversation/***the telehealth conversation ***increased my interest in biochemistry.***
- ***For my future studies, I would like to see more simulation-based teaching formats*** **(**like the telehealth conversation**).**
- **The communication exercises were a good preparation for the simulated conversation.**
- ***The roles and scenarios are clear and easy to understand.***
- The conversations with friends and family members during the self-learning phase made me feel well-prepared for the simulated conversation/the telehealth conversation.
- *The preparation for the simulated conversation/*the telehealth conversation *in **the group setting made me feel well-prepared for my role.***
- ***The simulated conversation/***the telehealth conversation ***put me in a realistic situation.***

- ***The feedback discussion that followed the simulated conversation*/the telehealth conversation *were helpful as well.***

The evaluation was based on grades from A (very good) to F (fail).

- ***I would grade the simulated conversation/*** the telehealth conversation ***including its preparation as follows.***

Free text

Below, you can provide feedback, either positive or negative; above all, specific suggestions for improvement regarding the simulated conversation and its preparation.

Reference

25. Schneider A, Messerer DAC, Kuhn V, Horneffer A, Bugaj TJ, Nikendei C, Kühl M, Kühl SJ. Randomised controlled monocentric trial to compare the impact of using professional actors or peers for communication training in a competency-based inverted biochemistry classroom in preclinical medical education. BMJ Open. 2022;12(5):e050823-050823. DOI: 10.1136/bmjopen-2021-050823
